# A comparison of smartphone and paper data-collection tools in the Burden of Obstructive Lung Disease (BOLD) study in Gezira state, Sudan

**DOI:** 10.1371/journal.pone.0193917

**Published:** 2018-03-08

**Authors:** Rana Ahmed, Ryan Robinson, Asma Elsony, Rachael Thomson, S. Bertel Squire, Rasmus Malmborg, Peter Burney, Kevin Mortimer

**Affiliations:** 1 The Epidemiological Laboratory, Khartoum, Sudan; 2 Liverpool School of Tropical Medicine, Liverpool, United Kingdom; 3 LHL's International Tuberculosis Foundation, Oslo, Norway; 4 Imperial College, London, United Kingdom; Chest Research Foundation, INDIA

## Abstract

**Introduction:**

Data collection using paper-based questionnaires can be time consuming and return errors affect data accuracy, completeness, and information quality in health surveys. We compared smartphone and paper-based data collection systems in the Burden of Obstructive Lung Disease (BOLD) study in rural Sudan.

**Methods:**

This exploratory pilot study was designed to run in parallel with the cross-sectional household survey. The Open Data Kit was used to programme questionnaires in Arabic into smartphones. We included 100 study participants (83% women; median age = 41.5 ± 16.4 years) from the BOLD study from 3 rural villages in East-Gezira and Kamleen localities of Gezira state, Sudan. Questionnaire data were collected using smartphone and paper-based technologies simultaneously. We used Kappa statistics and inter-rater class coefficient to test agreement between the two methods.

**Results:**

Symptoms reported included cough (24%), phlegm (15%), wheezing (17%), and shortness of breath (18%). One in five were or had been cigarette smokers. The two data collection methods varied between perfect to slight agreement across the 204 variables evaluated (Kappa varied between 1.00 and 0.02 and inter-rater coefficient between 1.00 and -0.12). Errors were most commonly seen with paper questionnaires (83% of errors seen) vs smartphones (17% of errors seen) administered questionnaires with questions with complex skip-patterns being a major source of errors in paper questionnaires. Automated checks and validations in smartphone-administered questionnaires avoided skip-pattern related errors. Incomplete and inconsistent records were more likely seen on paper questionnaires.

**Conclusion:**

Compared to paper-based data collection, smartphone technology worked well for data collection in the study, which was conducted in a challenging rural environment in Sudan. This approach provided timely, quality data with fewer errors and inconsistencies compared to paper-based data collection. We recommend this method for future BOLD studies and other population-based studies in similar settings.

## Introduction

Traditionally paper-based data collection has been the mainstay of data gathering for the Burden of Obstructive Lung Disease (BOLD) study. BOLD is an international recognized study that uses standardised methods to measure the burden of chronic obstructive lung disease [1]. However automated data collection and processing methods are becoming more widespread in healthcare research [2, 3] and they have many advantages [4–6]. There are studies investigating the use of automated data collection via smartphones as a research tool in developing countries however, it lacks the quantity available in the developed world and there are far less exploring its use in large-scale and complex surveys, such as BOLD [6].

Paper-based data collection is convenient for many researchers and data collectors. It has several potential advantages over the automated method; data extraction is not limited to a specific place and it is seemingly easier to produce, modify, manipulate, and implement. In addition, they can provide a long-lasting record of all modifications and an instant evaluation of forms can be completed by different review authors [7, 8]. Moreover, data loss using the paper-based method is potentially less likely than with automated data collection [7].

Studies from developing countries have found, however, that using paper-based methods result in a higher frequency of incomplete records, a greater potential for human errors, and more time is needed to organise the data [1, 7, 9, 10]. In addition, it can involve labour-intensive data entry and may limit timely analysis [10].

In the last decade, the number of mobile phone users in Africa has dramatically increased, with Africa now representing 83% of the total mobile phone subscribers worldwide. South Africa leads the region in mobile phone ownership with 36.4 mobile phones per 100 people. They are no longer considered a luxury [5, 6].

As information communication technologies grow and software such as the ‘Android’ systems platform and many open-source applications have been developed, researchers in the health sector have begun using smartphones as a tool in patient data collection, disease surveillance, clinical research, and national surveys [2, 7]. However, paper-based questionnaires continue to be the main data collection tool in many countries, especially in sub-Saharan Africa [7].

Using smartphone technology-based tools for data collection has many advantages and can provide a broader range of options. It is economically and environmentally friendly, and it can provide faster reporting with more accuracy [11]. It is also more efficient. Data collection and entry can be combined into one step [2], forms can be easily developed to provide built-in checking and reliability rules, and has additional features such as a Global Positioning System (GPS). In addition, time stamps, alarms, automatic completions, and reminders can help in work-rate monitoring and data validation [4, 8, 12].

In contrast, data security and connectivity can be a concern, and data collectors need to be familiar and comfortable with using an automated tool [4]. Accidental loss of data, battery life, loss or theft of the device, security of the device, and network connectivity in rural areas are also major concerns [5, 11, 13].

In Sudan, both the government and civil society frequently use smartphones for data gathering. There is, however, very little published research regarding this. Therefore, the main aim of this study is to compare accuracy, completeness, and quality of data collected using smartphone-based versus paper-based methodologies for the BOLD study in Gezira State, Sudan. We explored whether data collection might be expedited by conducting this work as a one-step process ‘in the field’ (smartphone-based data collection), improving the ability to have timely questionnaire data and be more responsive to the data in real time. In addition, we aim to provide evidence that will assist decision-making with regards to the method of data collection to use in future BOLD surveys in rural Sudan.

## Methods

### Study design and setting

The parent BOLD study is a cross-sectional household survey with a multi-stage random sampling plan, conducted in 35 villages in Gezira state, Sudan. This exploratory cross-sectional household survey ran within the same setting of the rural BOLD survey following the same study design and targeted the same population with identical inclusion criteria: all participants were non-institutionalised adults, aged 18 years and older, and living in Gezira state.

Convenience sampling of 100 accessible participants from 3 random villages was conducted between August and September 2016. Each of these villages was composed of 30 households, which were similar in all study characteristics including population density, education level, geographic area, sex distribution, ethnic groups, and infrastructure. The sample size was based on the feasibility of this pilot.

The method of data collection was randomized separate from the main BOLD survey. Firstly, a total of eight samples were randomized daily (four to be administered by smartphone and four by paper) prior to the start of data collection. The data collector administering the questionnaires for the BOLD team was then randomized. Two out of four teams from the main BOLD survey conducted data collection per day. The main BOLD survey core questionnaire data collection and the method of collection data then ran simultaneously.

Prior to the start of this study, a needs assessment was conducted with the aim of increasing data collection efficiency and to avoid errors that are common in household surveys. This was decided based on discussion with different experts including a software engineer, an epidemiologist, and a public health expert in health surveys. This exercise identified essential requirements to develop and implement the electronic data collection forms and system. A wide range of available software was explored. An ODK was selected based on affordability as an open source application, previous evidence-based use in developing countries, and the ability for offline data entry. Standard operating procedures (SOPs) in line with the ethical approval were developed prior to the start of data collection.

### Open Data Kit (ODK) questionnaire development

The ODK[14] was used by the study principle investigator to develop an electronic version of the BOLD core questionnaire for the Samsung S3 mobile phone, which includes six main sections: demographic information, respiratory symptoms and disorders (cough, sputum, wheezing, and shortness of breath), use of medication, cigarette smoking, occupational exposure, economic impact, and activity limitation. The questionnaire was composed of 26 pages with 44 questions (with multiple sub-questions and different skip patterns). Two-hundred four fields were to be completed for each questionnaire, consisting of 48 keyed-in, open-ended questions and 105 multiple-choice questions. A unique six-digit study ID was manually assigned to the participant (following BOLD study protocol) and all questionnaires were labelled with a serial number (i.e. 1–100).

Initially the ODK questionnaire was designed using Microsoft excel and then using guidance from the ODK instructions and xlsform.org, an XLSForm was developed. To avoid errors, validation check boxes, reliability rules, alerts, skip patterns, and fields requiring data were programmed into the ODK. The form had three different sheets, the first of which was a ‘survey sheet’, which included all collectable data in Arabic as well as the GPS location with start and end times of the survey. Notes to guide data collectors, including hints and data constraints where also added to this sheet. The second was a ‘choices sheet’, which contained a comprehensive list of all answer options with labels in Arabic and English. The third sheet, of ‘settings’, contained the form title in the mobile interface and the form ID.

Questions were arranged by group and answers were programed based on either being open ended or single/multiple choice questions. The excel file was then uploaded into http://opendatakit.org/xiframe/ and the full functioning form previewed in “Enketo” (a preview provided by the ODK). XML forms were then uploaded into ODK Aggregate. To use the forms on the mobile device the ODK collect application for android devices was used.

After development, the tool was tested and validated in one village separate from the two villages selected for the study. The data collection of smartphone-based forms was carried out by the study principal investigator and another trained data collector from BOLD team. Six data collectors collected the paper-based forms.

ODKs can work offline. As Internet connectivity was very limited in the study area, data were entered into smartphone during the collection, saved, and later uploaded to the server.

### Data collection and entry

Data collection comprised recording participants’ information simultaneously on paper and electronic forms. Two data collectors concurrently completed the core questionnaire alternatively in a random order (i.e. if the questionnaire was conducted using the paper-based method, the smartphone data collector must complete the smartphone-based form, and vice versa) to ensure no additional burden on participants. The order of administration of either the paper questionnaire or the smartphone followed the randomisation conducted daily. Study SOPs were agreed between the two administrators before the start of the data collection.

The paper-based questionnaire data was double entered in a predesigned SPSS (IBM Corp. Released 2015. IBM SPSS Statistics for Windows, Version 23.0. Armonk, NY: IBM Corp) data spreadsheet. It was entered into two different spreadsheets, and crosschecking and cleaning was performed before the analysis stage. Smartphone forms were submitted to the ODK aggregate server and then retrieved using Briefcase, saved as a CVS file, compiled, and then transformed into SPSS format.

All data collectors were graduates and had an extensive training of BOLD questionnaire administration, research methods and ethics by senior researchers, data manager and IT specialist. The mobile data collectors were additionally trained in how to use the ODK and how to administer the BOLD core questionnaire using the Samsung S3 mobile phones.

### Ethical considerations

Ethical approval from Liverpool School of Tropical Medicine Research Ethics Committee was obtained prior to start this study (Ref. 11.03RS). In addition, for the BOLD main study, ethical approval was obtained from the ethics committee at the Imperial College London and the research department at Ministry of Health in Gezira state, Sudan. During the data collection stage, all data were anonymised. All participants received information sheets regarding the study details and written consent was obtained to participate in this study.

### Statistical analysis

Descriptive statistics for the main variables in the questionnaire were performed. Frequency distributions and descriptive statistics were conducted to test data completeness. A chi squared test was used to test the association between the percentage of forms with errors and form type (smartphone-based/paper-based), for collection order (first versus second), and possible interaction of form type by order.

Inter-rater measurement agreement tests were used to check the agreement between continuous variables, while percentage agreement and Kappa statistics were used to test the agreement between the categorical variables. P-values of less than .05 were used to report the significance of the results. Kappa ranges are described in Table 1 [15].

**Table 1:** Kappa range and level of agreement.

Kappa range	Level of agreement
0	Less than chance
0.01–0.20	Slight
0.21–0.40	Fair
0.41–0.60	Moderate
0.61–0.80	Substantial
0.81–0.99	Almost perfect

## Results

### Study population demographics

Of the 100 participants (median age = 41.5 ± 16.4 years), 63% were women. Perfect agreement (inter-rater measurement agreement = 0.929) was shown between the two methods in the age variable (Table 2).

**Table 2:** Characteristics and demographic information of study participants.

Variable	Smartphone Frequency (%) (N = 100)	Paper Frequency (%) (N = 100)
**Age (years)**		
18–30	28 (28.0%)	28 (28.0%)
31–40	21 (21.0%)	21 (21.0%)
41–50	15 (15.0%)	15 (15.0%)
51–60	19 (19.0%)	19 (19.0%)
60–70	15 (15.0%)	13 (13.0%)
> 70	2 (2.0%)	4 (4.0%)
**Sex**		
Male	37 (37.0%)	38 (38.0%)
Female	63 (63.0%)	62 (62.0%)
**Education level**		
Primary school	26 (26.0%)	26 (26.0%)
Middle school	11 (11.0%)	11 (11.0%)
High school	28 (28.0%)	28 (28.0%)
College (trade/professional/ community)	6 (6.0%)	6 (6.0%)
Four-year college/university	22 (22.0%)	19 (19.0%)
None	6 (6.0%)	6 (6.0%)
Unknown	1 (1.0)	1 (1.0)
**Father education level**		
Primary school	15 (15.0%)	15 (15.0%)
Middle school	7 (7.0%)	7 (7.0%)
High school	6 (6.0%)	7 (6.0%)
College (trade/professional/ community)	4 (4.0%)	1 (1.0%)
Four-year college/university	9 (9.0%)	9 (9.0%)
None	28 (28.0%)	33 (33.0%)
Unknown	31 (31.0%)	28 (28.0%)
**Mother education level**		
Primary school	26 (26.0%)	29 (29.0%)
Middle school	6 (6.0%)	5 (5.0%)
High school	12 (12.0%)	11 (11.0%)
College (trade/professional/ community)	2 (2.0%)	2 (2.0%)
Four-year college/university)	2 (2.0%)	3 (3.0%)
None	36 (36.0%)	37 (37.0%)
Unknown	16 (16.0%)	13 (13.0%)

Two ethnic groups were dominant in the studied population: Halaween represented 42% while Jaalia represented 17%. Other ethnic groups were also reported. Kappa statistics showed substantial agreement between the two methodologies (Kappa = 0.630). Date of birth also represented a strong agreement. Generally, the two methodologies agreed on all demographic information on the questionnaire.

In ‘higher education level achieved’, two records were missing in paper-based data and inconsistencies in the values of both methods were seen; however, the Kappa statistics showed strong to substantial agreement.

### BOLD questionnaire results

The BOLD questionnaire results are summarised in Table 3.

**Table 3:** Respiratory symptoms, smoking, occupational exposure and economic impact for BOLD questionnaire (n = 100).

Variable	Number (%)
Cough often, without viral illness	24(24%)
Productive cough, without viral illness	15(15%)
Wheeze in the last 12 months	17(17%)
Shortness of breath	18(18%)
Took respiratory medicine in last 12 months	19(19%)
Ever smoked Cigarette	20(20%)
Current smoker	40(40%)
Ex-smoker	60(60%)
Ever smoked water pipe	6(6%)
Think smoking can cause serious illness	97(97%)-99(99%)
Worked in an occupation that has dust	20(20%)-30(30%)
Work for income generation	38(38%)-40(40%)
Unemployed	60(60%)-62(62%0
Unemployed because of respiratory problems	2(2%)
Unemployed because of other health problems	19(19%)

### Areas of strongest agreement

Several BOLD questionnaire areas showed almost perfect agreement between the two data collection methods. The major questions relating to cough had a 99% agreement, with one recorded missing in paper-based forms, and major questions relating to phlegm had 100% agreement between the two methods. Questions relating to emphysema, asthma, and chronic bronchitis all showed strong to perfect agreement, with two questions missing.

Strong to perfect agreement was also seen in smoking section as shown in Table 4. No significant differences were found on other smoking questions such as smoking *shisha*, cigars, *canapés*, and special substances. All other smoking related questions had a strong level agreement between the smartphone and paper records and substantial agreement was also found in the questions regarding smoking at work (kappa = 0.77, p < .05).

**Table 4:** Smoking.

Variable	Interclass coefficient	p-value	Interpretation
How old were you when you started smoking?	1.000^1^	p < .001	High inter-rater agreement
How old were you when you quit smoking?	1.000^1^	p < .001	High inter-rater agreement
How many cigarettes do you smoke daily?	0.976^2^	p < .001	High inter-rater agreement
How many cigarettes do you smoke weekly?	0.552^1^	0.025	Low inter-rater agreement

1 = perfect agreement

2 = almost perfect agreement.

Significant low-inter-rater agreement was found on questions related to the number of weekly smoked cigarettes (Interclass coefficient = 0.552, p < .05) (Table 4). The mobile variable was calculated automatically in the smartphone-based version and it was calculated manually in the paper-based one.

Absolute agreement was demonstrated in most of the questions regarding medication use (Table 5). The timeframe of taking the medication should have been specified in days and weeks in both methods of data collection. In paper-based questionnaires, there were no specifications for this timeframe (days/weeks); however, in the smartphone-based questions, this criterion was covered. Of the ten participants taking medications, only two had the timeframe of medication duration specified. Kappa values cannot be calculated for this variable, although there was 11% agreement between them.

**Table 5:** Medication.

Variable	Kappa statistic	p-value
Took medication in the past 12 months	0.935^2^	p < .001
Type of medication	.696^3^	p < .001
When was medication taken	.833^2^	p < .001
Period in months	1.000^1^	p < .001

1 = perfect agreement

2 = almost perfect agreement

3 = substantial agreement.

Occupation exposure showed strong agreement (kappa = 0.88) and inter-rater class statistic showed an absolute agreement between the two variables for a question related to ‘number of years of exposure’. Similarly, questions surrounding participant comorbidities also had a strong level of agreement between the two versions of the data, apart from questions regarding heart disease, which had fair agreement (kappa = 0.393).

Moderate to substantial agreement was found in questions regarding participant’s knowledge of smoking related disease (see Table 6).

**Table 6:** Knowledge, opinions, and attitudes.

Variable	Kappa statistic	p-value
Smoking causes stroke	0.421^4^	< .05
Smoking causes heart attack	0.550^4^	< .05
Smoking causes lung cancer	0.487^4^	< .05
Smoking causes chronic bronchitis	0.663^3^	< .05
Smoking causes emphysema/COPD	0.563^4^	< .05

3 = substantial agreement

4 = moderate agreement.

The sections regarding participant views of their health, how they feel, and how well they can do their usual activities showed strong agreement using Kappa statistics with values of 0.927 and 0.818. The only exception was a singular question regarding physical health and emotional problems. In this question, substantial agreement was shown (kappa = 0.795).

In questions addressing phlegm, a slight inconsistency was shown in the skipped pattern between the two methods. While fair level of agreement (kappa = 0.23, p < .05) was seen in skip-pattern questions relating to ‘hearing wheeze’ (Table 7).

**Table 7:** Respiratory symptoms and disorder.

Variable	Kappa statistic	p-value
Cough most days	1.000^1^	< .001
Phlegm most days	0.867^2^	.001
Wheeze in the past 12 month	0.895^2^	< .001
Wheeze only with cold	.239^5^	.309
Wheeze with shortness of breath	1.000^1^	< .001
Cannot walk because of shortness of breath	.858^2^	< .001
Shortness of breath when going uphill	-.013^7^	.895
Walk slower because of shortness of breath	.040 ^6^	.674
Stop walking to breath better	.111^6^	.439
Stop for breath after walking 100 yards	.149^6^	.170

1 = perfect agreement

2 = almost perfect agreement

5 = fair agreement

6 = slight agreement

7 = less than chance agreement.

### Areas of weakest agreement

The main areas of disagreement were in questions with skip patterns. In the ‘shortness of breath’ questions demonstrating disagreement, 15 of the paper-based questionnaires had used the skip pattern incorrectly (e.g. ‘if the answer is no, then the next two questions should be skipped’). Kappa statistics showed no agreement in the results from the two different methods in this area (Table 7). Where the skip questions properly followed strong agreement was found (kappa = 0.839).

The skip pattern was also not followed in questions related to ex-smokers. Four of 11 did not follow the skip pattern; however, these answers were correctly skipped for the same data in the smartphone questionnaires. Substantial agreement was found in these variables (kappa = 0.770, p < .05).

The same problem was encountered in the economic impact section, which showed variation in the level of agreement. The ‘skip pattern’ questions had moderate to substantial agreement, while the remainder had strong agreement (Tables 8 and 9).

**Table 8:** Economic impact (1).

Variable	Kappa statistic	p-value
**Work days lost**		
Did you work for income?	0.985^2^	< .05
Did you not work for income mainly due to breathing problems?	0.492^4^	< .05
Did you not work for income because you were a full-time homemaker or caregiver?	0.803^3^	< .05
Did your health problems stop you from performing your usual homemaking/caregiving tasks?	0.576^4^	< .05
During the past 12 months, did health problems stop you from working for income?	1.000^1^	< .05
**Non-work activities missed**		
Did health problems prevent you from participating in one or more non-work related activities?	0.758^3^	< .05
How many days did you not participate in non-work related activities due to your health problems?	1.000^1^	< .05
How many days did days you not participate in non-work related activities specifically due to breathing problems?	1.000^1^	< .05

1 = perfect agreement

2 = almost perfect agreement

3 = substantial agreement

4 = moderate agreement.

**Table 9:** Economic impact (2).

Variable	Interclass coefficient	p-value
How many days were you unable to perform your homemaking/caregiving tasks due to your health problems?	1.000^1^	< .05
During the past 12 months, how many days were you unable to perform your homemaking/caregiving tasks specifically due to breathing problems?	1.000^1^	< .05
How often during the past 12 months did you work for income?	0.997^2^	< .05
How many days were you unable to work for income due to your health problems?	-0.115^3^	.559

1 = perfect agreement

2 = almost perfect agreement

3 = Less than chance of agreement

### Incomplete records

Missing records were seen in different paper questionnaires in different questions as shown in (Table 10).

**Table 10:** Incomplete records in 100 questionnaires.

Questionnaire section	Number of missing records in Paper based forms	Number of missing records in Mobile based forms
**Demographics**	13	0
**Respiratory symptoms and disorderr**	11	0
**Medications**	5	0
**Smoking**	12	2
**Knowledge attitude and perception**	5	0
**Occupational exposure**	2	0
**Additional comorbidities**	9	0
**Views about your health**	1	0
**Economic**	13	0

### Forms with errors

Three variables of error were created based on (1) whether the form had an error, (2) the collection methodology containing the error, and (3) the data collector. Errors were defined as questions with no answers or wrong use of the ‘skip pattern’. In smartphone-based forms, most of the errors found were due to having different answer options (Table 11). A chi Square test demonstrated that paper forms were significantly more likely to have errors (82.5% of the total). In comparison, 10.5% occurred on smartphone-based questionnaires and 7% occurred in both formats (X^2^ (3, n = 100) = 64, p < .001). There was no significant association between having a form with errors and the order of administering the questionnaire using the smartphone or paper-based first (p = .686).

**Table 11:** Forms with returned errors.

Variable		Frequency (Percentage)	p-value (Pearson Chi-Square)	Correlation (Pearson's R)
**Error type**				
	Error in paper forms	47 (82.5%)	< .05	-.995
	Error in mobile forms	5 (10.5%)		
	Error in both	4 (7.0%)		
**Administration method**				
	Mobile based	27 (47.4%)	.686	0.061
	Paper based	30 (52.6)		
**Data Collector**				
	1	9 (15.8%)	.496	-.038
	2	16 (28.1%)		
	3	2 (3.5%)		
	4	8 (14.0%)		
	5	8 (14.0%)		
	6	14 (24.6%)		

There was no statistically significant association between the occurrence of errors in forms and the data collector (p = .496) and weak negative correlation between them (Pearson’s R = -0.038).

In addition, a multiple linear regression was calculated to predict the errors on forms based on who did the data collection and what order was used to administer the questionnaire; however, no significant association was found (F(2,97) = 0.299, p > .05, R^2^ = 0).

## Discussion

Data collection is one of the most important steps in conducting health research and having consistent, complete, and accurate data is vital to this. We found that the use of a smartphone-based data collection method in rural Sudan is feasible and provides timely and quality data with a lower number of errors and inconsistencies compared to data collected using paper-based methods. These findings are in line with other studies conducted in similar settings [3, 9, 12, 15, 16]. A recent study of routine influenza sentinel surveillance in Kenya and southern India both reported similar results, with less errors and inconsistencies in the smartphone collected data [2, 9].

Smartphone data collection provided more timely data for analysis in comparison to the paper-based forms. Paper collected data took around 15 days post initial collection to be double entered in SPSS software and cleaned, where as electronic data were rapidly accessible and retrieval took one day A collection date, start time, and end time of each questionnaire could also be electronically logged when using the smartphone, whereas this information was not routinely available for the paper-based method.

Although no cost-specific data were collected or evaluated in our study, we know that the electronic data collection had a low cost as we used two existing smartphones (Samsung Galaxy S3). Using an open source application, such as ODK, also decreased the electronic cost of this study.

The disagreements in data collection were mainly demonstrated in the questions with complicated skip patterns in mobile-based forms. However, the use of automated checks and validations in electronic forms prevented the occurrence of these errors, which was also found in a Kenyan influenza surveillance study [9]. Using the wrong skip pattern on such surveys can affect the quality of collected data and may lead to misleading results.

Variables without mandatory data entry requirements in mobile-based forms and open-ended questions caused 8 (80%) of the errors from a total of 10 returned errors in the mobile-based forms. Other studies reported similar findings with the limitation of unrestricted questions on mobile data collection [9]. The remaining 20% were due to technical programming problems.

Some of the reported disagreements may not have been caused by the data collection method used, but rather because the participant may not have provided clear answers. During the data collection, it was dependent on the researcher to choose the option he/she understands. Therefore, the disagreement in questions (views about your health—how you feel and how well you can do your usual activities) may not have been caused by the collection method used. Similarly, when assessing the level of higher education, there were differences in the ‘unknown’ and ‘none’ classifications as this was dependent on the researcher’s personal assessment.

### Challenges, limitations, and lessons learnt

One of the main limitation of this study was having a convenience sampling plan and a sample size of 100 participants. The sample size was selected based on the number of previous recruited participants within the main survey per month as well as time and budget constraints. In addition, because of the length of BOLD questionnaires the interviews could only be performed once per individual. Ideally the questionnaires would be taken using both methodologies simultaneously by the same data collector to allow more comparisons regarding the administration time and efficiency of data collectors.

Although the ODK tool is an open source application, some obstacles were faced during the development and data extraction stages as well as during the retrieval stage. First, the accessibility of the aggregate website in Sudan was challenging. During data collection, the ODK application would occasionally and unpredictably close and the researcher would need to restart the application. As the Internet connection and network coverage was very limited in the three rural villages, the availability of offline data collection in ODK makes it a viable tool in rural settings.

Second, using the Arabic language in the ODK software was challenging during questionnaire development and data retrieval, especially concerning the open-ended questions. Importing the CVS files from ODK into Excel was an issue as Excel was not able to interpret the Arabic content of the CVS file correctly.

One skip pattern in the smoking section had a programming error, which in turn created errors in two of the mobile forms as the options were incorrectly skipped.

The main challenge to both the paper and electronic forms was the length of the BOLD core questionnaire and the frequent skip patterns. The duration affects the programming time, collection, data entry and analysis in the electronic version as well as being a challenge for the data collectors. They were experts in using the paper based core questionnaire and sometimes faster than the smartphone-based data collectors when explaining the questions to participants. In future studies, further data collector experience may accelerate smartphone-based data collection [2].

Choosing a single part of the questionnaire for smartphone data collection would increase the speed of obtaining data and would demonstrate the effectiveness of electronic data collection as clearly. However, this method would still remain applicable for longer health surveys, which has been supported by past research [5, 16].

**Fig 1:**
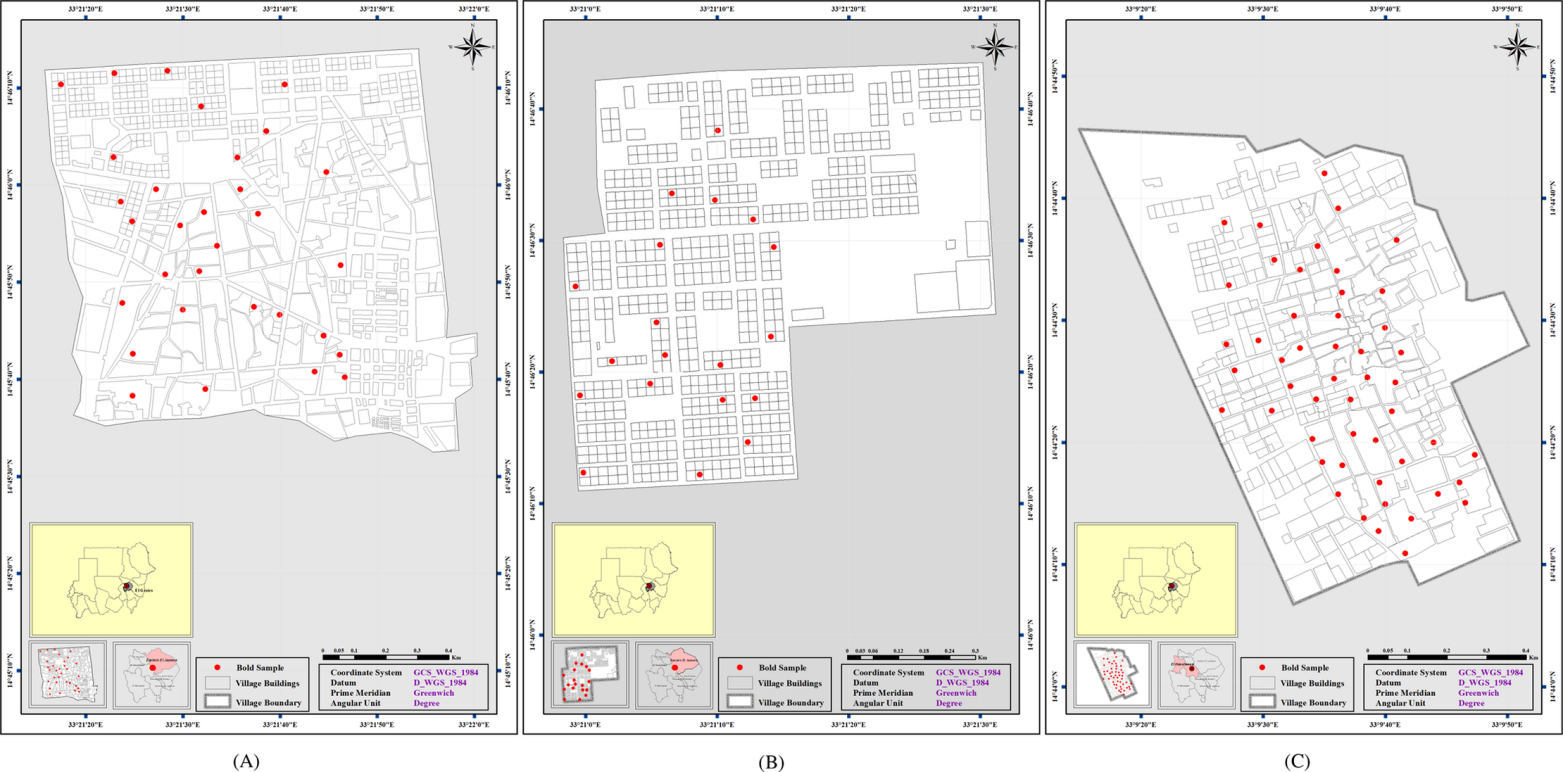
Maps showing geographical locations of study participants in included villages in Gezira state, Sudan. (A) Rufaa Alhai 4 village. (B) Rufaa Alhai 20 village. (C) Altakala Joubara village.

The electronic format in theory allows for the GPS coordinates to be easily obtained during the interview process and therefore accurate mapping of the study participant’s location, even in rural areas. However, in practice the GPS was variable, especially in the first village and there was a delay in obtaining data. If this is fully functional, it does represent a potential advantage over traditional paper-based data collection (Fig 1).

The overall economic cost regarding the use of mobile data collection method, including the software development, mobile/electronic devices, network connection, and internet accessibility, needs further investigation. The use of local servers rather than using cloud servers provided by the ODK platform also needs evaluating.

Lastly, for large questionnaires with many nested questions and multiple skip patterns such as BOLD core questionnaire, having either mobile version questionnaire with strict validation checks or minimising the skip patterns in paper based forms may improve the data collection process and improve the data quality.

## Conclusions

This study demonstrates that electronic data collection via smartphones for the BOLD study is an effective and efficient method in the challenging environment of rural Sudan. It provides timely and accurate data that is comparable with the traditional paper-based formats, with a lower level of user error. When disagreements between the two formats did occur, this was mainly due to the inappropriate completion of the BOLD skip-format questions in paper-based questionnaires. While the smartphone based technique is not without challenges, this study adds to the growing body of evidence for electronic data collection as a feasible method of obtaining data for health surveys in similar rural environments.

## Supporting information

S1 Dataset Merged mobile and paper based collected data for BOLD core questionnaire. (SAV)

S1 Permission Letter Maps permission letter. (JPG)

## References

[1] Buist AS, Vollmer WM, Sullivan SD, Weiss KB, Lee TA, Menezes AMB, et al The Burden of Obstructive Lung Disease Initiative (BOLD): rationale and design. COPD J Chronic Obstr Pulm Dis. 2005;2(2): 277–283. doi: 10.1081/COPD-576102 17136954

[2] Garg S, Mony PK. Electronic data capture for health surveys in developing countries: use of a mobile phone based application in southern India. Indian Journal of Medical Informatics. 2013 6 30;7(2):84–93.

[3] Thriemer K, Ley B, Ame SM, Puri MK, Hashim R, Chang N, et al Replacing paper data collection forms with electronic data entry in the field: findings from a study of community-acquired bloodstream infections in Pemba, Zanzibar. BMC Res Notes. 2012;5(1): 113 Available from: http://www.biomedcentral.com/1756-0500/5/113. 22353420 10.1186/1756-0500-5-113 PMC3392743

[4] King C, Hall J, Banda M, Beard J, Bird J, Kazembe P, et al Electronic data capture in a rural African setting: evaluating experiences with different systems in Malawi. Glob Health Action. 2014;7(4): 25878 Available from: http://www.pubmedcentral.nih.gov/articlerender.fcgi?artid=4216812&tool=pmcentrez&rendertype=abstract. 25363364 10.3402/gha.v7.25878 PMC4216812

[5] Tomlinson M, Solomon W, Singh Y, Doherty T, Chopra M, Ijumba P, et al The use of mobile phones as a data collection tool: a report from a household survey in South Africa. BMC Med Inform Decis Mak. 2009;9: 51 doi: 10.1186/1472-6947-9-51 20030813 10.1186/1472-6947-9-51 PMC2811102

[6] Schuster C, Brito CP. Cutting costs, boosting quality and collecting data real-time–Lessons from a cell phone-based beneficiary survey to strengthen Guatemala’s conditional cash transfer program. The world bank/EnBreve. 2011 Available from: http://siteresources.worldbank.org/INTLAC/Resources/257803-1269390034020/EnBreve_166_Web.pdf.

[7] King JD, Buolamwini J, Cromwell EA, Panfel A, Teferi T, Zerihun M, et al A novel electronic data collection system for large-scale surveys of neglected tropical diseases. PLoS One. 2013;8(9): e74570 Available from: http://dx.plos.org/10.1371/journal.pone.0074570. doi: 10.1371/journal.pone.0074570 24066147 10.1371/journal.pone.0074570 PMC3774718

[8] Higgins JP, Green S. Cochrane handbook for systematic reviews of interventions (Version 5). John Wiley & Sons. Available from: www.cochrane-handbook.org.

[9] Njuguna HN, Caselton DL, Arunga GO, Emukule GO, Kinyanjui DK, Kalani RM, et al A comparison of smartphones to paper-based questionnaires for routine influenza sentinel surveillance, Kenya, 2011–2012. BMC Med Inform Decis Mak. 2014;14(1): 107 Available from: http://www.biomedcentral.com/1472-6947/14/107. 25539745 10.1186/s12911-014-0107-5 PMC4305246

[10] Weber BA, Yarandi H, Rowe MA, Weber JP. A comparison study: paper-based versus web-based data collection and management. Appl Nurs Res. 2005;18(3): 182–185. Available from: http://linkinghub.elsevier.com/retrieve/pii/S089718970500039X. doi: 10.1016/j.apnr.2004.11.003 16106337 10.1016/j.apnr.2004.11.003

[11] Pakhare AP, Bali S, Kalra G. Use of mobile phones as research instrument for data collection. Indian Journal of Community Health. 2013 8 21;25(2):95–8.

[12] Le Jeannic A, Quelen C, Alberti C, Durand-Zaleski I. Comparison of two data collection processes in clinical studies: electronic and paper case report forms. BMC Med Res Methodol. 2014;14(1): 7 Available from: http://www.biomedcentral.com/1471-2288/14/7. 24438227 10.1186/1471-2288-14-7 PMC3909932

[13] Yu P, de Courten M, Pan E, Galea G, Pryor J. The development and evaluation of a PDA-based method for public health surveillance data collection in developing countries. Int J Med Inform. 2009;78(8): 532–542. Available from: http://www.biomedcentral.com/1472-6947/14/107. doi: 10.1016/j.ijmedinf.2009.03.002 19369114 10.1016/j.ijmedinf.2009.03.002

[14] Brunette W, Sundt M, Dell N, Chaudhri R, Breit N, Borriello G. Open data kit 2.0: expanding and refining information services for developing regions. InProceedings of the 14th Workshop on Mobile Computing Systems and Applications 2013 2 26 (p. 10). ACM.

[15] Isara AR, Onyeagwara NC, Lawin H, Irabor I, Igwenyi C, Kabamba L. Survey of airflow obstruction in two African countries: paper questionnaire versus mobile phone technology. African J Resirotry Med. 2013;8(2): 13–16.

[16] Dillon DG, Pirie F, Rice S, Pomilla C, Sandhu MS, Motala AA, et al Open-source electronic data capture system offered increased accuracy and cost-effectiveness compared with paper methods in Africa. J Clin Epidemiol. 2014;67(12): 1358–1363. Available from: http://linkinghub.elsevier.com/retrieve/pii/S0895435614002388. doi: 10.1016/j.jclinepi.2014.06.012 25135245 10.1016/j.jclinepi.2014.06.012 PMC4271740

