# Supplementary figures and images for: A comparison of smartphone and paper data-collection tools in the Burden of Obstructive Lung Disease (BOLD) study in Gezira state, Sudan
Source: PLoS One. 2018 Mar 8;13(3):e0193917. doi: 10.1371/journal.pone.0193917 (PMC5843227; doi:10.1371/journal.pone.0193917)

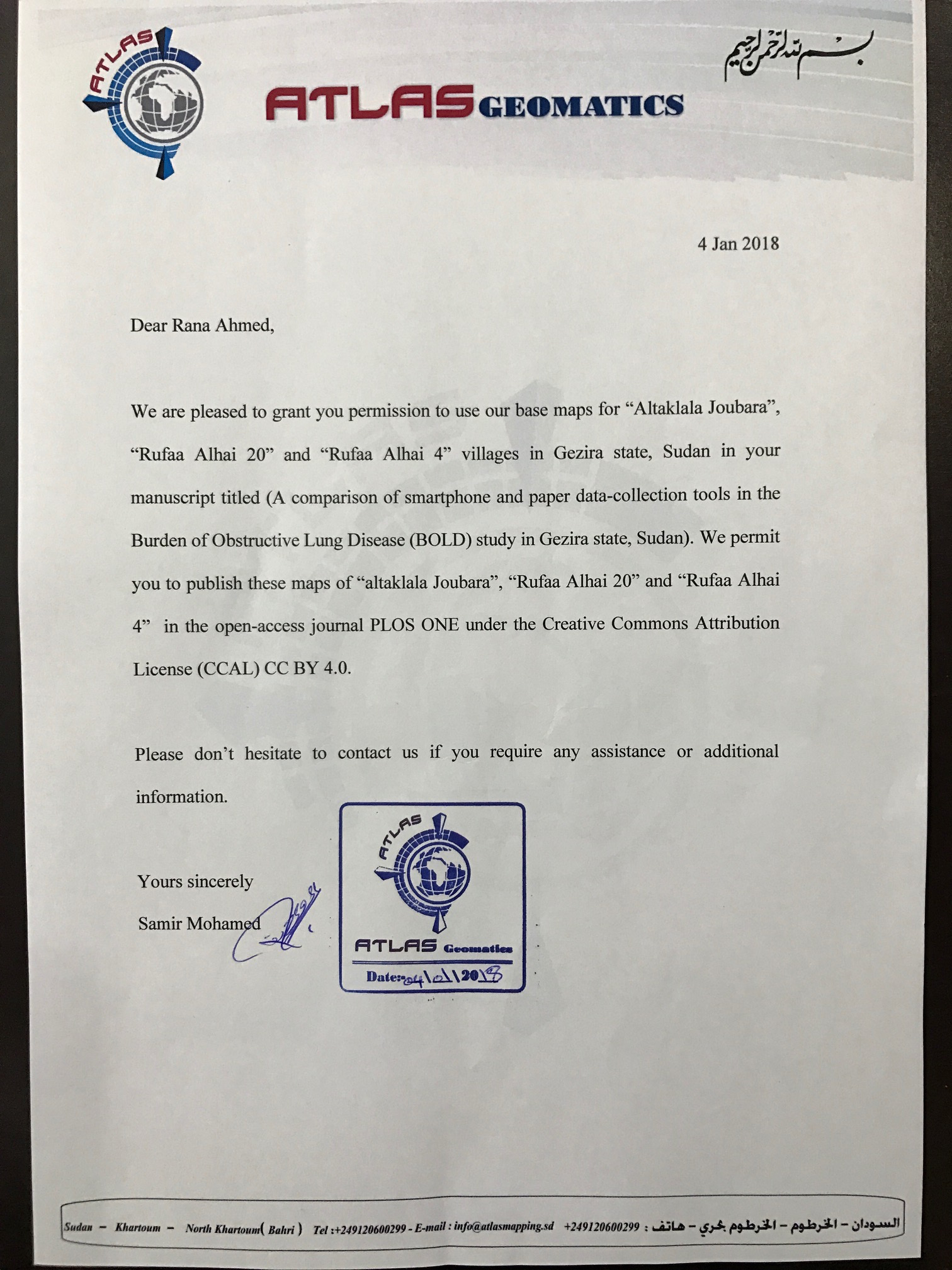

Supplement: S1 Permission Letter — (JPG) [file pone.0193917.s002.jpg]
